# Tumour response interpretation with new tumour response criteria *vs* the World Health Organisation criteria in patients with bone-only metastatic breast cancer

**DOI:** 10.1038/sj.bjc.6605546

**Published:** 2010-01-26

**Authors:** T Hamaoka, C M Costelloe, J E Madewell, P Liu, D A Berry, R Islam, R L Theriault, G N Hortobagyi, N T Ueno

**Affiliations:** 1 Department of Stem Cell Transplantation and Cellular Therapy, The University of Texas M. D. Anderson Cancer Center, Houston, TX, USA; 2 Breast Cancer, St Luke's International Hospital, Tokyo, Japan; 3 Department of Diagnostic Radiology, The University of Texas M. D. Anderson Cancer Center, Houston, TX, USA; 4 Department of Biostatistics and Applied Mathematics, The University of Texas M. D. Anderson Cancer Center, Houston, TX, USA; 5 Department of Breast Medical Oncology, The University of Texas M. D. Anderson Cancer Center, Houston, TX, USA

**Keywords:** bone diseases, breast neoplasms, diagnostic imaging, response assessment

## Abstract

**Background:**

We compared the utility of a new response classification (MDA; based on computed tomography (CT), magnetic resonance imaging (MRI), plain radiography (XR), and skeletal scintigraphy (SS)) and the World Health Organisation response classification (WHO; based on XR and SS) in stratifying breast cancer patients with bone-only metastases with respect to progression-free survival (PFS), overall survival (OS), and clinical response.

**Methods:**

We retrospectively reviewed 41 patients with bone-only metastatic breast cancer and assigned responses according to the MDA and WHO criteria. We analysed whether the MDA or WHO response classifications correlated with PFS and OS.

**Results:**

With the MDA criteria, there were significant differences in PFS between patients classified as responders and those classified as nonresponders (*P*=0.025), but with the WHO criteria, there were not. Neither criteria distinguished responders from nonresponders in terms of OS. MDA response criteria correlated better than WHO response criteria with clinical response assessment.

**Conclusions:**

The MDA classification is superior to the WHO classification in differentiating between responders and nonresponders among breast cancer patients with bone-only metastases. Application of the MDA classification may allow bone lesions to be considered measurable disease. Prospective study is needed to test the MDA classification among patients with bone metastasis.

Bone is one of the most common sites to which breast cancer metastasises (Coleman and Rubens, 1987; Hortobagyi, 1991). Up to 85% of patients with bone metastasis have other visceral metastases during the course of the disease (Coleman and Rubens, 1987; Hortobagyi, 1991). Skeleton-related events such as bone pain or pathologic fractures can substantially reduce the quality of life for long-term survivors (Johnson *et al*, 2003). Standard treatments for bone metastasis are anticancer agents, such as chemotherapy and endocrine therapy. Bisphosphonates are also used to prevent skeleton-related events. Response to treatment is typically estimated by using a combination of methods, including diagnostic imaging, measurement of biochemical markers, and evaluation of patients’ symptoms.

Imaging modalities such as plain radiography (XR), skeletal scintigraphy (SS), computed tomography (CT), magnetic resonance imaging (MRI), and positron emission tomography (PET) can be used to assess the response of bone lesions to treatment. However, a comprehensive strategy for assessing bone tumour response with these modalities is lacking, in part because of the complex interactions between tumour cells and host cells during bone turnover or remodeling. The presence of metastatic lesions from breast cancer can influence bone homeostasis to favour bone resorption or bone formation by affecting the activity of osteoclasts or osteoblasts, thereby resulting in osteolytic, osteoblastic, or mixed lesions. Thus, accurate assessment of the response of bone metastases to treatment requires visualising not only the tumour burden but also structural changes in the bone. Each of the aforementioned imaging techniques has advantages and disadvantages in this regard. With XR, currently the most convenient and inexpensive way of assessing treatment response, 3–6 months and >30–50% mineral loss may be required before changes become visible (Bellamy *et al*, 1987; Howell *et al*, 1988). In addition, although XR can depict changes in bone structure, it cannot depict the tumour itself. With SS, which reflects bone blastic activity, it can also take 6 months or longer to reliably detect a response because of the confounding effect of the flare phenomenon, a spurious increase in radionuclide uptake because of reparative mineralisation around healing metastases (Coleman, 1991; Hortobagyi, 1991). Although CT is not commonly used to scan the whole body, CT can depict both structural changes in the bone and anatomic changes associated with the target tumour because of its multiple window settings. Magnetic resonance imaging is optimally suited for showing spinal cord status and changes in the bone marrow but not suited to showing lytic or blastic change in bone structure. FDG PET, which reflects high glucose metabolism, can be used to assess bone tumour response in osteolytic metastatic lesions (Du *et al*, 2007), but it lacks the detail necessary to detect anatomic changes in response to treatment. Positron emission tomography–computed tomography is a new method of combining metabolic and anatomic information, but it is not yet commonly available for routine screening (Even-Sapir, 2005). Thus, proper assessment of the response of bone metastases to treatment requires consideration of different aspects of the lesions, use of diagnostic imaging modalities in appropriate combinations, and use of accurate, standardised response criteria. Unfortunately, the complexities of this process have led to the practise of considering bone lesions unmeasurable disease.

Computed tomography and MRI are now commonly used in clinical practise to assess bone tumour response, but the evidence of an advantage of these imaging modalities over conventional XR or SS is limited (Hamaoka *et al*, 2004). Until recently, no published response criteria included findings from diagnostic CT or MRI. The two established sets of criteria for assessing bone tumour response, one from the International Union Against Cancer (UICC) (Hayward *et al*, 1977) and the other from the World Health Organisation (WHO, 1979), are 30 years old and based on findings from XR or SS, which, as explained earlier, are limited in that 6 months or more may be needed before responses become visible. These criteria are not adequate as they do not incorporate modern methods (e.g., CT and MRI) of assessing the response of bone metastatic lesions to treatment. Another system in broad use, the Response Evaluation Criteria in Solid Tumours classification (Therasse *et al*, 2000), does not include bone lesions in response assessments. Therefore, in the absence of established response criteria, current response assessments for bone lesions using CT or MRI are highly dependent on the physician's judgment.

There is an urgent need to develop appropriate criteria for assessing tumour response in the bone because patients with bone-only metastatic disease have traditionally been excluded from clinical trials owing to the lack of such criteria. Further, the existence of an objective method with which community physicians can evaluate their patients in a timely manner and determine the effectiveness of treatment in eliciting a response in bone metastases may affect the quality of the care provided to these patients.

We hypothesised that CT or MRI is more accurate in assessing the response of bone metastatic lesions to treatment than is XR or SS because CT and MRI can visualise both bone and tumour and, presumably, changes in both that are associated with treatment (Hamaoka *et al*, 2004). To test this hypothesis, we compared the ability of our ‘MDA classification’, which takes into account findings from CT and/or MRI (Hamaoka *et al*, 2004) (Table 1), and the WHO classification, which does not, to stratify breast cancer patients with bone-only metastases with respect to progression-free survival (PFS), overall survival (OS), and clinical response.

## Materials and methods

This study was approved by the institutional review board of The University of Texas MD Anderson Cancer Center. We identified 46 patients with breast cancer and bone-only metastases who were observed at MD Anderson Cancer Center and given systemic treatment from October 1991 to September 2004 and who had CT, XR, SS, and/or MRI examinations available for review (a total of 180 imaging examinations). We excluded the patients who were given bisphosphonates because such treatment might affect the appearance of bone on imaging studies. Five of the 46 patients were not included in the statistical analysis because they underwent additional treatment before the first response assessment 2–6 months after the initiation of systemic therapy. All patients participated in clinical trials or standardised treatment protocols and received systemic therapy (chemotherapy in 34 patients and endocrine therapy in 7 patients). As this study focused on comparing imaging response criteria and diagnostic imaging before and after treatment, the results should not be affected by the type of treatment or chemotherapy regimen. Images were obtained at baseline (before the start of systemic therapy) and at 2–6 months after the beginning of systemic therapy and/or at 11–13 months after the beginning of systemic therapy. The broad time ranges for the two response assessment points were a result of the retrospective nature of this study. The timing of follow-up bone imaging commonly changed during the course of the study according to the tumour and treatment status.

All images were reviewed and responses assigned independently by two board-certified radiologists who specialise in musculoskeletal radiology (CMC, JEM) and who were blinded to patient identities and outcomes. A response was assigned to each imaging study (XR, SS, CT, or MRI). In addition, a response was assigned to each patient on the basis of each of the three sets of imaging response criteria (UICC, WHO, and MDA) (Table 1). The UICC criteria are based only on findings from XR, the WHO criteria include XR and SS, and the MDA criteria include findings from XR, SS, CT, and MRI (Table 1). Therefore, XR images were read three times – once in terms of the UICC criteria, again in terms of the WHO criteria, and a third time in terms of the MDA criteria. Skeletal scintigraphy images were read twice, in terms of the WHO and MDA criteria. Computed tomography and MRI scans were read only once, in terms of the MDA criteria. Responses were categorised as complete response, partial response, stable disease, or progressive disease. In total, 431 separate assessments (180 image sets) were made by each radiologist. Final responses were confirmed by consensus, with discrepant diagnoses resolved through discussion by the two readers in the presence of a third investigator. Clinical evidence of response was obtained from evaluation of (1) symptom changes, (2) trends in the levels of tumour markers, and (3) all available radiographic images. If all three criteria showed stable disease or if one or more criteria showed disease progression, the findings were interpreted as indicating no response. If one or more criteria showed response and the others were stable, the findings were interpreted as indicating a response.

To verify the advantage of the MDA criteria, we analysed whether the use of a particular imaging modality or response classification would distinguish responders from nonresponders in terms of PFS or OS by using Kaplan–Meier analyses. To analyse which particular imaging modality or response classification most accurately reflected true bone tumour response, we analysed agreement between the response assigned on the basis of imaging results (response assigned on the basis of XR, SS, CT, or MRI alone and response assigned according to the UICC, WHO, and MDA criteria) and clinical response (complete or partial response *vs* stable or progressive disease) using McNemar's test and the kappa coefficient test.

The retrospective nature of the data collection prevented our obtaining enough XR and MRI scans for statistical analysis of XR or MRI as single modalities. Less number of XR synchronised images for each part by part, in turn precluded us from studying the UICC criteria, which are based only on XR. Less number of synchronised MRI is caused, which it is not standard to assess bone tumour response. Therefore, the role of MRI was precluded from the analysis. In addition, few image sets were available from the later assessment time (at 11–13 months after treatment). Therefore, we compared CT *vs* SS (to compare diagnostic imaging) and the MDA classification, which includes CT, XR, and SS, *vs* the WHO classification, which includes XR and SS, for the period between baseline and 2–6 months after treatment had begun.

## Results

### Patient characteristics

Patients’ characteristics are shown in Table 2. The median age at diagnosis was 42 years (range, 31–61 years). We did not attempt to separate patients into lytic and blastic subgroups because most bone metastases had both lytic and blastic components. The clinical response rates at 2–6 months after treatment initiation and at 11–13 months after treatment initiation were 36.6 and 30.6%, respectively. Only one patient died before 12 months after treatment initiation. The median follow-up period was 37 months.

### CT *vs* SS

**Figure 1:**
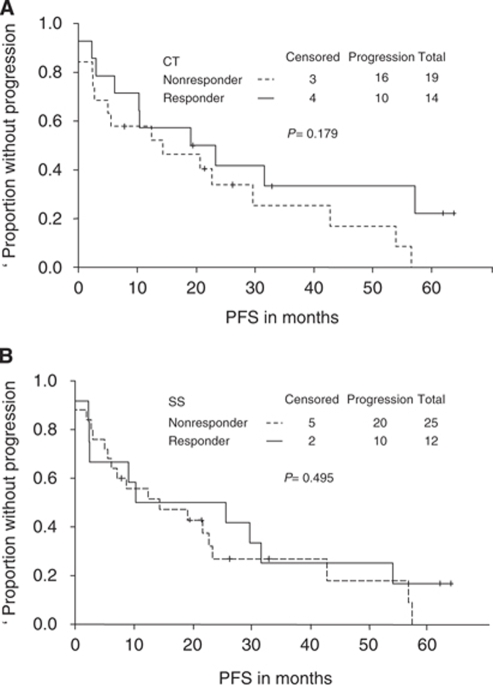
Progression-free survival (PFS) curves for patients who responded to treatment (complete or partial response) and those who did not (stable or progressive disease) according to computed tomography (CT) (**A**) or skeletal scintigraphy (SS) (**B**). CT seemed to distinguish responders from nonresponders during the first 6 months after treatment according to PFS, but SS did not.

Skeletal scintigraphy alone did not distinguish responders from nonresponders in terms of either PFS (median time to progression, 10.3 months for responders *vs* 14.3 months for nonresponders, *P*=0.50; Figure 1B) or OS (median survival time, 61.9 months for responders *vs* 59.9 months for nonresponders, *P*=0.80). Computed tomography alone also did not distinguish responders from nonresponders in terms of either PFS (median time to progression, 19.1 months for responders *vs* 14.3 months for nonresponders, *P*=0.18; Figure 1A) or OS (median survival time, 61.9 months for responders *vs* 34.4 months for nonresponders, *P*=0.38).

However, CT alone tended to correlate better than SS alone with true clinical response during the first 2–6 months after treatment (kappa coefficients, 0.44 and 0.05, respectively; McNemar's *P*=0.74 and 0.62, respectively; Table 3).

### MDA classification *vs* WHO classification

**Figure 2:**
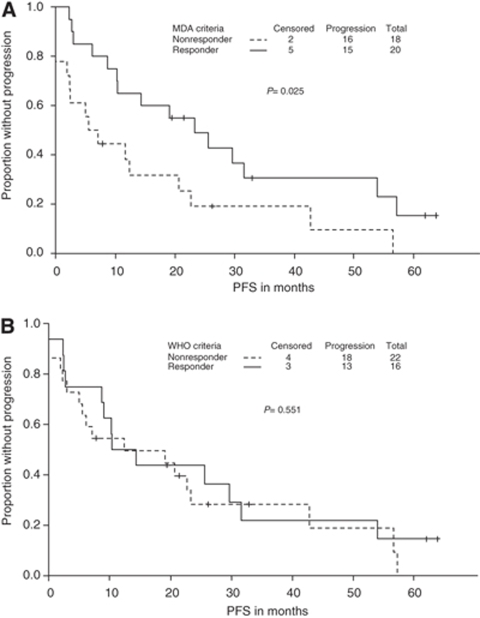
Progression-free survival (PFS) curves for patients who responded to treatment (complete or partial response) and those who did not (stable or progressive disease) according to the MDA criteria (**A**) and the World Health Organisation (WHO) criteria (**B**). The MDA criteria (which incorporate findings from CT scans) distinguished responders from nonresponders during the first 6 months after treatment according to PFS; in other words, patients classified as responders according to the MDA criteria (which included CT findings) had a better prognosis than did those classified as nonresponders. In contrast, the WHO classification (based on SS findings) did not differentiate between responders and nonresponders in terms of PFS.

The MDA classification, which includes SS and CT, distinguished responders from nonresponders in terms of PFS (median time to progression, 23.3 months for responders *vs* 5.5 months for nonresponders; *P*=0.025; Figure 2A). There was also a trend for difference between responders and nonresponders in terms of OS, but this difference was not significant (median survival time, 61.9 months for responders *vs* 34.4 months for nonresponders; *P*=0.13).

In contrast, the WHO classification did not distinguish responders from nonresponders in terms of either PFS (median time to progression, 12.4 months for responders *vs* 10.4 months for nonresponders; *P*=0.55; Figure 2B) or OS (median survival time, 61.9 months for responders *vs* 59.9 months for nonresponders; *P*=0.97). The MDA criteria tended to correlate better than the WHO criteria with true clinical response during the first 2–6 months after treatment (kappa coefficients, 0.53 and 0.07; McNemar's *P*=0.09 and 0.81, respectively; Table 3).

## Discussion

**Figure 3:**
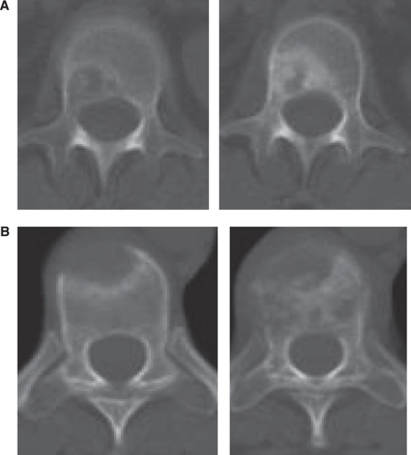
Computed tomography scans assessed with the MDA criteria. (**A**) Sclerotic change (right) in the rim of an originally lytic lesion (left) indicates a partial response. (**B**) Lytic progression (right) of an originally lytic lesion (left) indicates progressive disease.

We previously reported a new set of response criteria, the MDA criteria, that address the shortcomings of the UICC and WHO criteria by taking into account CT and MRI findings (Hamaoka *et al*, 2004) (Table 1). The MDA criteria also include detailed descriptions of anatomic changes to be considered for each diagnostic imaging modality. The MDA criteria take into account the fact that the structure of bone rarely heals such that the bone has the same appearance as the original even if treatment was significantly effective (complete response). For example, according to the MDA criteria, recalcification of the rim of an osteolytic lesion on XR or CT (Figure 3A) is considered partial response, and an increase in the area of lysis (Figure 3B) is considered progressive disease.

The results of this retrospective image reading analysis indicate that the MDA classification is superior to the WHO classification in differentiating between responders and nonresponders among breast cancer patients with bone-only metastases. With the MDA classification, which takes into account CT findings, there were significant differences in PFS between patients classified as responders and those classified as nonresponders within 2–6 months after treatment. With the WHO classification, which does not take into account CT findings, there were no such differences. The MDA criteria tended to be more sensitive than the WHO criteria for detecting response, although the number of cases studied was too few to permit definitive conclusions on this point. Computed tomography may be more sensitive than SS for discerning responses.

Few reports are available documenting survival outcomes according to response assessed using different response-assessment schemes. One study showed that survival rates among patients with ‘stable’ bone disease for more than 6 months according to the UICC criteria were similar to those among patients with a ‘partial response’ (Howell *et al*, 1988). In other words, in that study, the UICC criteria did not distinguish between responders and nonresponders in terms of survival rates.

Despite the widespread use of CT for assessing tumour response of solid non-bone tumours, the use of CT for assessing bone tumour response has yet to be established. We did find one published prospective study in which CT was used to assess the response of lytic metastatic bone lesions in 20 patients and CT response was compared with change in patients’ symptoms (Bellamy *et al*, 1987). In this study, improvement observed on CT was associated with improvement in symptoms in two-thirds of the patients. This study has shown that patients with a response on CT may have had longer PFS and OS than those who did not show a response.

The lower correlation between the primarily SS-based WHO criteria and response than between the MDA criteria and response could have resulted from several factors, including high false-positive rates caused by conditions other than tumour (e.g., fracture, arthritis, infection) (Galasko and Doyle, 1972; Citrin *et al*, 1977; Coleman *et al*, 1988b; Tubiana-Hulin, 1991; Rybak and Rosenthal, 2001) or ‘flare’ phenomena (Coleman, 1991; Hortobagyi, 1991). In one prospective report, in 75% of patients with breast cancer whose bone metastases showed a partial response (healing of lytic metastases on XR), there was increased tracer uptake on SS during the first 3 months after treatment because of new bone that had formed during the repair process. Such a situation could well be interpreted as progressive disease; however, after 6 months, the accumulation gradually decreased (Coleman *et al*, 1988a). Another possible explanation for the poor correlation between WHO response and clinical response is that rapid progression of disease, when overwhelming destruction allows little chance for new bone to form, is sometimes depicted by SS as a reduction in isotope uptake (‘cold spots’) (Galasko and Doyle, 1972; Condon *et al*, 1981; Cook and Fogelman, 2000). In our opinion, determining the final response of bone metastases solely on the basis of changes in radionuclide uptake over time is not appropriate (Libshitz and Hortobagyi, 1981).

Our findings suggest that the MDA criteria, which incorporate findings from CT scans, are superior to the WHO criteria, which are based primarily on SS, for predicting PFS in patients who respond to treatment. This confirms the importance of using multiple imaging modalities to accurately determine response. We found a statistically significant difference only for the MDA-to-WHO comparison for PFS. However, we speculate that the lack of significant differences in other comparisons may have been because of the limited number of patients who had multiple image sets available for retrospective image reviews. To increase the likelihood of identifying patients who had had imaging performed at regular intervals after treatment, we selected patients who had been treated according to the structured, well-organised treatment plans required of investigative research protocols; this necessarily limited the number of patients and images available for review.

In summary, the results of this image reading study suggest that the MDA classification is superior to the WHO classification for assessing the response of bone metastases to treatment. However, these findings need to be confirmed prospectively. In addition, in future prospective studies, the effect of bisphosphonates should be studied because bisphosphonates are now commonly used in patients with bone metastasis. Further, PET–CT, which fuses images from PET and CT, may yield better detection of bone tumour response because of the addition of information on glucose metabolism to the anatomic details provided by CT. One study showed 80.5% agreement between CT osteoblastic response and PET positivity change (positive to negative) in osteolytic metastases (Du *et al*, 2007). Positron emission tomography also has the advantage of permitting quantification of response – bone tumour response might be quantified using the maximum standard uptake value (Stafford *et al*, 2002). That indicates that PET can be a sensitive modality for monitoring osteolytic bone tumour response. Although PET scanning has the potential to yield false-positive results with the use of granulocyte colony-stimulating factors in patients receiving myelosuppressive chemotherapy, the CT aspect of PET–CT may compensate for that potential problem with its anatomic information (Even-Sapir, 2005; Israel *et al*, 2006). As the cost of PET–CT gradually becomes more reasonable, there is a great possibility that PET–CT may become the standard for assessing the response of bone metastasis.

## Figures and Tables

**Table 1:** The UICC, WHO, and MDA criteria for detection of bone response

**Response type**	**Union International Against Cancer (UICC)^a^**	**World Health Organisation (WHO)^b^**	**Revised criteria for assessment of bone response (MDA)**
Target diagnostic imaging	XR	XR, SS	XR, SS, CT, MRI
Complete response	Disappearance of all known disease Lytic lesions should have radiologic evidence of calcification	Complete disappearance of all lesions on X-ray or scan for at least 4 weeks	Complete fill-in or sclerosis of lytic lesion on XR and CT Disappearance of hot spots or tumour signal on SS, CT, or MRI Normalisation of osteoblastic lesion on XR and CT
Partial response	At least 50% decrease in size of measurable lesions Objective improvement in evaluable or unmeasurable lesions No new lesions or progressive lesions	Partial decrease in size of lytic lesions, recalcification of lytic lesions, or decreased density of blastic lesions for at least 4 weeks	Sclerotic rim about initially lytic lesion or sclerosis of lesions previously undetected on XR or CT Partial fill-in or sclerosis of lytic lesion on XR or CT Regression of measurable lesion on XR, CT, or MRI Regression of lesion on SS (exclude rapid regression^c^) Decrease in blastic lesion on XR or CT
No change or stable disease	Unchanged, or between 25% increase and 50% decrease in size of measurable lesions^d^	As a result of the slow response of bone lesions, the classification of ‘no change’ should not be applied until at least 8 weeks have passed from start of therapy	No change in measurable lesion on XR, CT, or MRI No change in blastic lesion on XR, CT, or MRI No new lesion on XR, SS, CT, or MRI
Progressive disease	Mixed; some lesions persist while others progress, or new lesions appear Failure; some or all lesions progress and/or new lesions appear. No lesions regress	Increase in size of existing lesions or appearance of new lesions	Increase in size of any existing measurable lesions on XR, CT, or MRI New lesion on XR, SS (excluding flare phenomena), CT, or MRI Increase in activity on SS (excluding flare phenomena) or blastic/lytic lesion on XR or CT

Abbreviations: SS= skeletal scintigraphy; XR= plain radiography; CT= computed tomography; MRI= magnetic resonance imaging.

a Criteria are based on plain radiography; the duration of response is to be measured from the start of therapy until either new lesions appear or any one existing lesion increases by 25% or more above its smallest recorded size.

b Occurrence of bone compression or fracture and its healing should not be used as the sole indicator for evaluation of therapy.

c Rapid osteolytic progression may show decreased osteoblastic activity, resulting in regression of ‘hot spots’ on SS. XR or CT may be helpful in detecting progressive osteolysis and thus helping to identify progressive disease in this situation.

d If lesions that cannot be measured but are otherwise evaluable represent the bulk of disease and these lesions clearly do not respond even though measurable lesions have improved, then the response is considered no change rather than an objective regression.

**Table 2:** Patients’ characteristics

**Characteristic**	**Number (%)**
No. of patients	41
Age, median (range)	42 years (31–61 years)
	
*Disease stage*	
I	7 (17)
II	21 (51)
III	4 (10)
IV	8 (20)
Unknown	1 (2)
	
*T status*	
1	11 (27)
2	24 (59)
3	2 (5)
4	3 (7)
Unknown	1 (2)
	
*N status*	
Positive	27 (66)
Negative	13 (32)
Unknown	1 (2)
	
*Estrogen receptor status*	
Positive	27 (66)
Negative	13 (32)
Unknown	1 (2)
	
*Progesterone receptor status*	
Positive	24 (59)
Negative	16 (39)
Unknown	1 (2)
	
*Her2/*neu *status*	
Positive	5 (12)
Negative	13 (32)
Unknown	23 (56)
	
*Bone metastatic site*	
Spine	36 (88)
Pelvis	18 (44)
Rib	14 (34)
	
*Type of treatment*	
Chemotherapy	34
Endocrine therapy	7
	
*Availability of images*	
At baseline and at 2–6 months after treatment initiation	40
XR images	13
SS images	37
CT images	34
MRI images	13
At baseline and at 11–13 months after treatment initiation	25
XR images	11
SS images	22
CT images	24
MRI images	12

Abbreviations: XR= plain radiography; SS= skeletal scintigraphy; CT= computed tomography; MRI= magnetic resonance imaging.

**Table 3:** Agreement between imaging responses and clinical response

	**Clinical response**			
**Imaging response**	**Non- responder**	**Res- ponder**	**McNemar's test *P*-value**	**Kappa coefficient +95% Conf. Limit**
*MDA*				
Nonresponder	16	2	0.09	0.53 (0.27, 0.79)
Responder	7	13		
				
*WHO*				
Nonresponder	14	8	0.81	0.07 (–0.24, 0.39)
Responder	9	7		
				
*CT*				
Nonresponder	15	4	0.74	0.44 (0.13, 0.75)
Responder	5	9		
				
*SS*				
Nonresponder	16	9	0.62	0.05 (–0.27, 0.38)
Responder	7	5		

Abbreviations: CT= computed tomography; SS= skeletal scintigraphy.
